# Training Family Medicine Residents in Dermoscopy Using an e-Learning Course: Pilot Interventional Study

**DOI:** 10.2196/56005

**Published:** 2024-05-13

**Authors:** Pauline Friche, Lionel Moulis, Aurélie Du Thanh, Olivier Dereure, Claire Duflos, Francois Carbonnel

**Affiliations:** 1 University Department of Family Medicine University of Montpellier Montpellier France; 2 Clinical Research and Epidemiology Unit Department of Public Health Montpellier University Hospital Montpellier France; 3 Pathogenesis and Control of Chronic and Emerging Infections University of Montpellier, Institut national de la santé et de la recherche médicale, Etablissement français du sang, University of Antilles Montpellier France; 4 Department of Dermatology Montpellier University Hospital Montpellier France; 5 Department of Dermatology University of Montpellier Montpellier France; 6 Department of Public Health University of Montpellier Montpellier France; 7 Desbrest Institute of Epidemiology and Public Health, Unité Mixte de Recherche, Unité d'accueil 11 University of Montpellier, Institut national de la santé et de la recherche médicale Montpellier France; 8 University Multiprofessional Health Center Avicenne Montpellier France

**Keywords:** dermoscopy, dermatoscope, dermatoscopes, dermatological, skin, training, GP, family practitioner, family practitioners, family physician, family physicians, general practice, family medicine, primary health care, internship and residency, education, e-learning, eLearning, dermatology, resident, residency, intern, interns, internship, internships

## Abstract

**Background:**

Skin cancers are the most common group of cancers diagnosed worldwide. Aging and sun exposure increase their risk. The decline in the number of dermatologists is pushing the issue of dermatological screening back onto family doctors. Dermoscopy is an easy-to-use tool that increases the sensitivity of melanoma diagnosis by 60% to 90%, but its use is limited due to lack of training. The characteristics of “ideal” dermoscopy training have yet to be established. We created a Moodle (Moodle HQ)-based e-learning course to train family medicine residents in dermoscopy.

**Objective:**

This study aimed to evaluate the evolution of dermoscopy knowledge among family doctors immediately and 1 and 3 months after e-learning training.

**Methods:**

We conducted a prospective interventional study between April and November 2020 to evaluate an educational program intended for family medicine residents at the University of Montpellier-Nîmes, France. They were asked to complete an e-learning course consisting of 2 modules, with an assessment quiz repeated at 1 (M1) and 3 months (M3). The course was based on a 2-step algorithm, a method of dermoscopic analysis of pigmented skin lesions that is internationally accepted. The objectives of modules 1 and 2 were to differentiate melanocytic lesions from nonmelanocytic lesions and to precisely identify skin lesions by looking for dermoscopic morphological criteria specific to each lesion. Each module consisted of 15 questions with immediate feedback after each question.

**Results:**

In total, 134 residents were included, and 66.4% (n=89) and 47% (n=63) of trainees fully participated in the evaluation of module 1 and module 2, respectively. This study showed a significant score improvement 3 months after the training course in 92.1% (n=82) of participants for module 1 and 87.3% (n=55) of participants for module 2 (*P*<.001). The majority of the participants expressed satisfaction (n=48, 90.6%) with the training course, and 96.3% (n=51) planned to use a dermatoscope in their future practice. Regarding final scores, the only variable that was statistically significant was the resident’s initial scores (*P*=.003) for module 1. No measured variable was found to be associated with retention (midtraining or final evaluation) for module 2. Residents who had completed at least 1 dermatology rotation during medical school had significantly higher initial scores in module 1 at M0 (*P*=.03). Residents who reported having completed at least 1 dermatology rotation during their family medicine training had a statistically significant higher score at M1 for module 1 and M3 for module 2 (*P*=.01 and *P*=.001).

**Conclusions:**

The integration of an e-learning training course in dermoscopy into the curriculum of FM residents results in a significant improvement in their diagnosis skills and meets their expectations. Developing a program combining an e-learning course and face-to-face training for residents is likely to result in more frequent and effective dermoscopy use by family doctors.

## Introduction

Skin cancers are the most common group of cancers diagnosed worldwide [1]. The number of new cases of cutaneous melanoma per year is expected to increase by more than 50% from 2020 to 2040 [1]. In the European Union, skin melanoma accounted for 4% of all new cancers diagnosed in 2020 and 1.3% of all deaths due to cancer [2]. Risk factors are individual (light phototype, age, male sex, personal history of skin cancer or predisposing skin lesions, and family history of melanoma) and environmental (prolonged sun exposure, artificial tanning, immunosuppression, and lower socioeconomic class) [3]. If detected early, these skin cancers have a good prognosis. Skin inspection is based on the ABCDE rule. It was the first dermoscopy algorithm used to differentiate benign from malignant melanocytic lesions [4]. Specifically, A stands for asymmetry—one half of the spot is unlike the other half. B stands for border—the spot has an irregular, scalloped, or poorly defined border. C stands for color—varying colors from one area to the next, such as shades of tan, brown or black, or areas of white, red, or blue. D stands for diameter, melanomas are usually greater than 6 mm but can be smaller. E is for evolving (the spot looks different from the rest or is changing in size, shape, or color). The ABCDE rule has a sensitivity ranging from 84% to 93% [3]. Dermoscopy relies on a handheld microscope and incident light to reveal images of the lower skin layers at magnifications of 10 to 100 times. It is said to increase the sensitivity of melanoma diagnosis by 60% to 90% [3].

In France, melanomas are traditionally diagnosed in 2 steps: a skin inspection by the family doctor (FD), during which the family doctor decides whether to refer the patient to a dermatologist, and a second stage carried out by a dermatologist, during which the decision is made whether to biopsy the lesion, based on skin inspection and dermoscopy. In total, 2981 dermatologists were active in 2023 in France (ie, 3.4 dermatologists per 100,000 inhabitants, with significant regional disparities) [5]. This is also the case for family doctors, but by 2025, their numbers could rise again. The number of dermatologists will continue to fall and is expected to reach 2.4 dermatologists per 100,000 inhabitants in 2030. Only 8% of French family doctors use a dermatoscope as part of their routine practice, compared with 40% in Australia, a country hit by a rise in melanoma [6,7]. The main obstacles limiting the use of dermatoscopes are the difficulty of accessing them in consultation and lack of training [8]. Proper training is even more important as this technique offers nothing more than skin inspection alone if the user is untrained or inexperienced [9,10]. It also improves FDs’ diagnostic accuracy and long-term performance, increases their confidence, reduces the propensity to refer benign lesions to a dermatologist, and improves the benign-to-malignant ratio of excised lesions [11].

The characteristics of “ideal” dermoscopy training have yet to be established [3]. They range from a 1-hour lecture to an intensive week-long course, often combined with digital tools [3]. It is acknowledged that e-learning is as effective as traditional teaching methods. However, there is still relatively little literature to attest to its general superiority [12]. The use of e-learning is becoming increasingly ubiquitous, especially among doctors who have recently graduated and since the COVID-19 pandemic [13,14]. Another advantage of e-learning is that it can be offered in asynchronous form, enabling participants to follow the course at their own pace. Incorporating a core training course in the family medicine (FM) specialty training program could enable these future family doctors not only to acquire dermoscopy knowledge and skills but also to become aware of the value of its use in their future practice. A survey of FM residency program directors in the United States revealed that only 6.8% of residents had 4 or more hours of dermoscopy training and asked for research to better understand how to facilitate dermoscopy training in family medicine residencies [15].

The Department of Faculty of Medicine at University of Montpellier-Nîmes designed an optional e-learning module to train second- and third-year family medicine residents in dermoscopy in 2020.

The primary objective of this study was to evaluate the evolution of dermoscopy knowledge among participants immediately after e-learning training and 1 and 3 months afterward. The secondary objectives were to determine whether sociodemographic characteristics or student satisfaction influenced e-learning results.

## Methods

### Study Design

This was a monocentric, nonrandomized, prospective interventional study carried out between April and November 2020. It was based on an assessment quiz hosted on Moodle (Moodle HQ), administered before (M0) and immediately after asynchronous distance training, with the same quiz administered at 1 month (M1) and 3 months (M3) after training. This format is in line with the learning theory known as the forgetting and recovering hypothesis, according to which forgetting makes for better learning [16].

The percentages of progression and regression between M0 and M1, M1 and M3, and M0 and M3 were calculated. The primary end point was the score at different time points (M0, M1, and M3). Score progression or regression was calculated by comparing scores over time. Students progressed if the difference in scores was strictly greater than 0 and regressed if the difference in scores was strictly less than 0.

There were several secondary end points: factors associated with information retention at M1 and M3 for both modules, factors associated with scores, and factors associated with satisfaction.

### Study Population

All family medicine residents who chose this optional course before April 2020 were included. The exclusion criteria were being a first-year resident (optional courses are offered only to the second or third year of the family medicine residency program). As there is an absence of an equivalent training course published in the literature, there was no basis for comparison for calculating the number of participants required. We chose to include the entire cohort of participants in this training.

### Intervention Description

**Figure 1:**
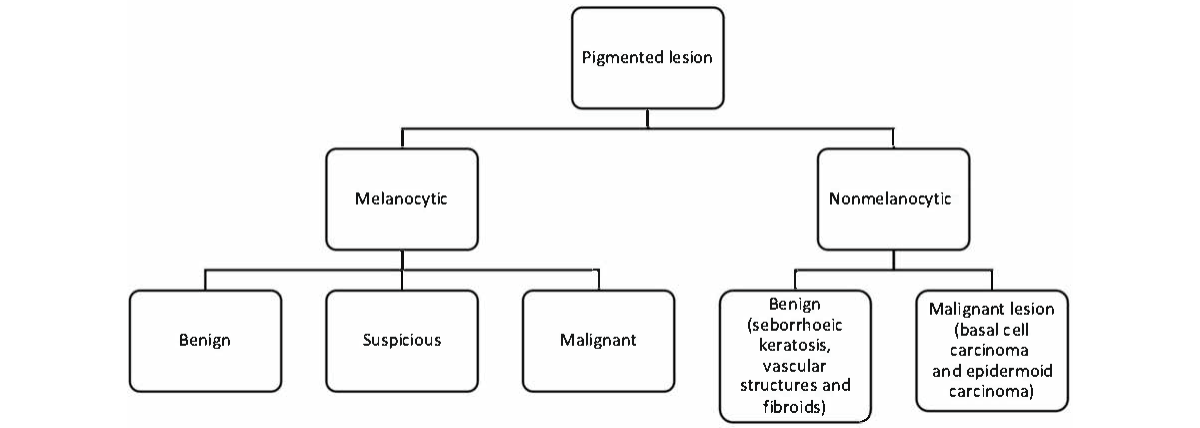
The 2-step algorithm.

The e-learning course was created between December 2019 and March 2020 by PF, FC, ADT, and OD. The course was divided into 2 modules with different levels of difficulty, in accordance with the 2-step algorithm [17]. This is a method of dermoscopic analysis of pigmented skin lesions that has been internationally accepted since 2001 (Figure 1). In module 1, “Dermoscopy level 1,” participants learned to differentiate melanocytic lesions from nonmelanocytic lesions. In module 2, “Dermoscopy level 2,” participants learned to precisely identify skin lesions by looking for dermoscopic morphological criteria specific to each lesion and to consider their specific management according to the nature of these lesions.

**Figure 2:**
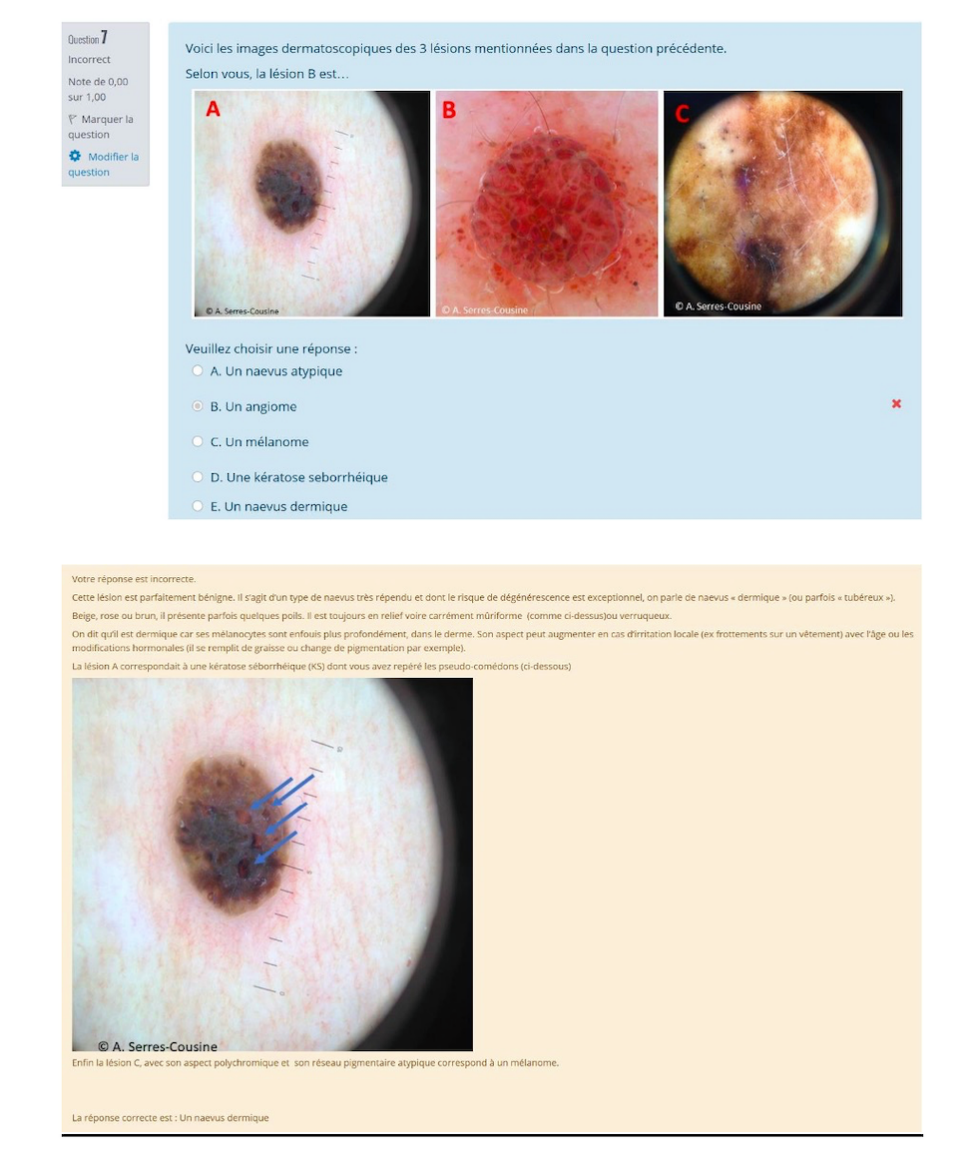
Sample multiple choice questions.

Each module consisted of 15 questions in the form of multiple choice, drag and drop, or short open-response questions. Each module was scored out of 15, with 1 point per question. Each question was set as a single attempt, with immediate feedback after each question. The feedback was accompanied by course notes (Figure 2). These modules served as assessment quizzes at all stages of the study. Each session lasted 15 minutes. Participants were given 3 weeks to complete each questionnaire. The images used in the clinical cases were anonymized and were freely provided by fellow dermatologists; the name of the doctor holding the image was cited.

Participants were asked to complete a prestudy survey: sex, year of family residency program, dermatology clinical rotation experience, locums in family medicine, dermatology training during medical school or family medicine residency, quality of dermatology training during family medicine residency, interest in dermatology, frequency of dermatological consultations, self-evaluation of dermatological skills and ability to use a dermatoscope. After completing the final post-test at M3, participants were invited to complete a satisfaction questionnaire. A “free comments” section was provided at the end of the questionnaire.

The course was originally designed to include a face-to-face workshop on using dermatoscopes and performing biopsies, following the e-learning modules, as part of a blended-learning approach [18]. However, due to the COVID-19 pandemic and the university shutdown, these workshops were canceled, and training videos were made available to participants upon completion of the satisfaction questionnaire.

### Statistical Analysis

Residents’ sociodemographic characteristics were described using mean and SD for continuous variables and frequency and percentages for categorical ones. Residents’ mean scores were compared regarding their characteristics using 1-tailed Student *t* tests. To evaluate the differences in scores between the different time points, Friedman tests were performed. To analyze the factors associated with knowledge retention, we used a multivariate linear regression after checking the normality of the variable (score variations). Independent variables were sociodemographic and pathway data selected by stepwise procedures with an entry *P* of .20 and an exit *P* of .15.

The satisfaction score was the sum of two 4-item Likert scales assessing training evaluation and practical application. The satisfaction score theoretically ranged from 2 to 8. No linear regression was applicable because of its nonlinearity. Instead, we performed a median split and evaluated the factors associated with a satisfaction score greater than or equal to 7. A logistic regression model was used in which the independent variables were the sociodemographic and academic variables and the scores at the different temporalities after the selection of the variables by a stepwise procedure with an entry *P* of .20 and an exit *P* of .15. For the free comments section, we analyzed the verbatim of the satisfaction response in free text manually, comment by comment. Postcodification was carried out using a lexical approach, and percentages of response modalities were calculated. All analyses were performed with SAS (version 9.4; SAS Institute) using 2-tailed statistical tests with α set at 5%.

### Ethical Considerations

The need for consent was deemed unnecessary according to national regulations. Research into changes in practices brought about by training medical or paramedical staff for research purposes is considered noninterventional in France and therefore does not require an ethical opinion [19]. Still, the information provided at the start of the e-learning course indicated that, by taking part in the course, the participants were consenting to participate in this study. The images used in the course were anonymized. Participant data were also anonymized. They were not compensated and took part voluntarily in this program, which was valued as part of their general medicine curriculum.

## Results

### Participants

**Figure 3:**
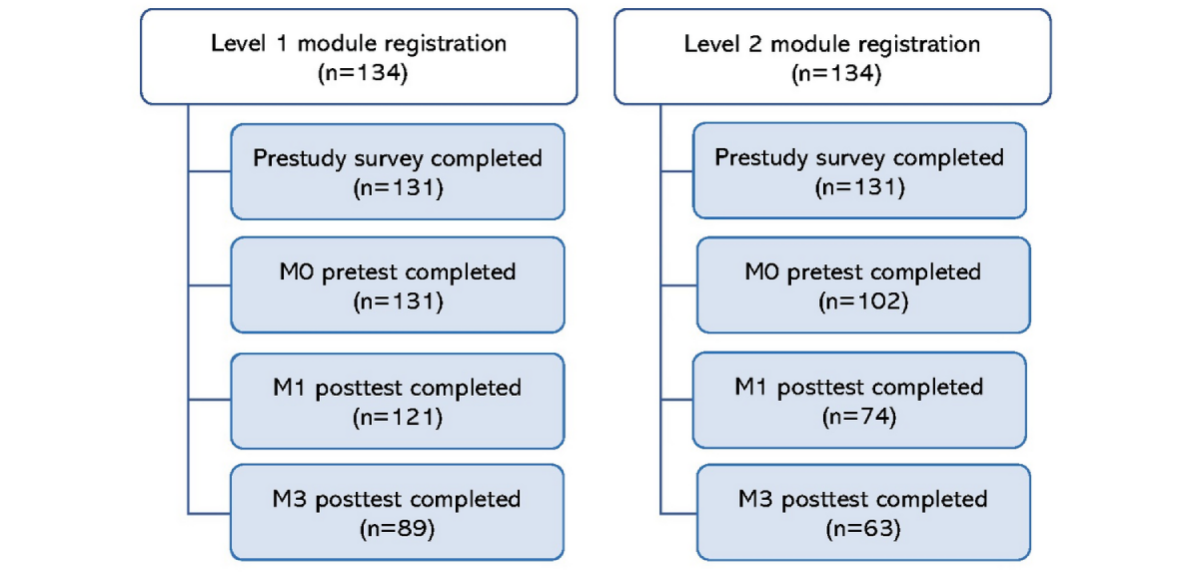
Flowchart of study participation.

A flowchart details study participation (Figure 3). The study included a total of 134 residents. Eighty-nine (66.4%) and 63 (47%) trainees participated in the full evaluation (M0, M1, and M3) of module 1 and module 2, respectively. Their sociodemographic characteristics are detailed in Table 1.

Residents who reported having completed at least 1 dermatology rotation during medical school had significantly higher initial scores in module 1 at M0 than those who had not (*P*=.03). This was not the case for the other assessment periods or module 2. Residents who reported having completed at least 1 dermatology rotation during their FM training had a statistically significant higher score at M1 for module 1 and M3 for module 2 (*P*=.01 and *P*=.001). Residents who were interested in dermatology had a higher score on M1 for module 1 (*P*=.01). Residents who reported frequently seeing patients for dermatologic reasons had higher scores on M3 for module 1 (*P*=.03).

Regarding the final scores, the only variable that was statistically significant was the resident’s initial scores (*P*=.003) for module 1. No measured variable was found to be associated with retention, either at midtraining or at the final evaluation for module 2.

**Table 1:** The sociodemographic characteristics of the participants (n=134) and respective statistical comparisons for each module and assessment time point.

Sociodemographic characteristics			Participants, n (%)		Level 1 module score at each month, mean (SD)							Level 2 module score at each month, mean (SD)			
					0		1		2		0		1		2
**Sex**															
	Male	43 (32.8)		10.85 (1.55)		12.63 (1.24)		13.31 (1.27)		8.23 (1.84)			10.12 (1.59)	11.18 (1.89)	
	Female	88 (67.2)		10.81 (1.82)		12.56 (1.43)		13.27 (1.35)		9.09 (2.13)			10.19 (2.03)	11.08 (1.97)	
	*P* value^a^			.89		.76		.88		*.04*			.85	.84	
**Year of FM^b^ residency program**															
	Second year	66 (50.4)		10.74 (1.85)		12.62 (1.48)		13.28 (1.25)		8.59 (2.29)			9.85 (1.91)	10.73 (2.15)	
	Third year	65 (49.7)		10.94 (1.41)		12.55 (1.26)		13.28 (1.4)		9.02 (1.84)			10.51 (1.85)	11.56 (1.57)	
	*P* value^a^			.49		.78		.98		.3			.12	.053	
**Hospital dermatology rotation during medical school**															
	Yes	50 (38.2)		11.21 (1.41)		12.85 (1.28)		13.41 (1.28)		9.22 (1.98)			10.40 (1.95)	11.29 (1.65)	
	No	81 (61.8)		10.61 (1.74)		12.40 (1.4)		13.19 (1.35)		9.56 (2.1)			10.03 (1.88)	10.99 (2.11)	
	*P* value^a^			*.03*		.07		.42		.11			.41	.48	
**6-month hospital dermatology rotation during FM residency**															
	Yes	18 (13.7)		11.44 (1.49)		13.18 (0.94)		13.68 (0.95)		9.6 (1.6)			11.03 (1.76)	12.52 (1.24)	
	No	113 (86.3)		10.74 (1.65)		12.48 (1.4)		13.21 (1.37)		8.67 (2.12)			10 (1.89)	10.88 (1.94)	
	*P* value^a^			.08		*.01*		.12		.054			.07	*.001*	
**Locums in FM during FM residency**															
	Yes	35 (26.9)		10.91 (1.58)		12.35 (1.22)		13.00 (1.52)		8.95 (2.05)			10.43 (1.66)	11.33 (1.14)	
	No	95 (72.5)		10.81 (1.68)		12.72 (1.35)		12.38 (1.23)		8.75 (2.1)			10.03 (1.98)	11.00 (2.1)	
	*P* value^a^			.77		.16		.26		.66			.38	.39	
**Use of the dermatoscope**															
	Acquired	26 (19.8)		10.83 (1.47)		12.71 (1.11)		13.54 (1.14)		8.81 (2.05)			10.2 (2.38)	11.43 (1.91)	
	Not acquired	105 (80.2)		10.84 (1.69)		12.55 (1.43)		13.21 (1.36)		8.82 (2.09)			10.16 (1.79)	11.03 (0.95)	
	*P* value^a^			.98		.53		.29		.98			.95	.48	
**Interest in dermatology**															
	Very interested	36 (27.5)		10.58 (1.74)		12.43 (1.34)		13.06 (1.58)		9.25 (2.72)			9.78 (1.56)	11.38 (1.77)	
	A little interested	80 (61.1)		10.91 (1.53)		12.82 (1.31)		13.38 (1.22)		8.74 (1.72)			10.29 (2.01)	11.22 (2.09)	
	Not really interested	15 (11.4)		11.98 (2.01)		11.63 (1.4)		13.27 (1.26)		8.29 (1.93)			10.26 (1.97)	10.38 (1.39)	
	Not interested at all	0 (0)		0 (0)		0 (0)		0 (0)		0 (0)			0 (0)	0 (0)	
	*P* value^a^			.50		*.01*		.63		.33			.62	.33	
**Frequency of dermatological consultations**															
	Often (at least 1 a day)	35 (26.7)		10.34 (1.75)		12.51 (1.64)		12.69 (1.59)		8.12 (2.46)			10.04 (1.48)	11.00 (1.25)	
	Regularly (at least 1 a week)	93 (71)		11.02 (1.58)		12.66 (1.24)		13.5 (1.13)		9.04 (1.86)			10.22 (2.07)	11.17 (2.19)	
	Seldom (less than 1 a week)	3 (2.3)		11.2 (1.14)		11.12 (1.04)		14.09 (0.83)		9.75 (3.42)			10.33 (2.12)	10.61 (1.25)	
	*P* value^a^			.11		.15		*.02*		.12			.92	.88	
**Dermatology skills**															
	Fairly comfortable	34 (26)		10.72 (1.43)		12.6 (1.28)		13.51 (0.99)		8.74 (1.87)			9.86 (1.81)	10.95 (2.15)	
	In difficulties	86 (65.6)		10.82 (1.79)		12.64 (1.42)		13.23 (1.43)		8.94 (2.22)			10.47 (1.92)	11.28 (1.94)	
	Not comfortable at all	11 (8.4)		11.38 (0.85)		12.1 (1.25)		13.1 (1.29)		8.16 (1.28)			8.81 (1.44)	10.5 (1.51)	
	*P* value^a^			.5		.48		.65		.53			.06	.48	
**Evaluation of dermatology training in medical school**															
	Practice oriented	56 (42.8)		10.6 (1.67)		12.72 (1.36)		13.2 (1.1)		9.04 (2.11)			10.06 (1.84)	10.98 (2.22)	
	Not adapted to practice	75 (57.2)		11.02 (1.61)		12.47 (1.37)		13.34 (1.46)		8.66 (2.05)			10.26 (1.96)	11.20 (1.74)	
	*P* value^a^			.16		.33		.61		.37			.64	.65	
**Evaluation of dermatology training during residency**															
	Practice-oriented	18 (13.7)		10.46 (1.52)		12.22 (1.62)		13.47 (1.07)		9.09 (1.78)			10.22 (2.14)	10.79 (2.34)	
	Not adapted to practice	113 (86.3)		10.9 (1.66)		12.64 (1.32)		13.25 (1.36)		8.78 (2.12)			10.16 (1.88)	11.16 (1.88)	
	*P* value^a^			.27		.30		.5		.57			.4	.2	
**Training satisfaction**															
	Very satisfactory	34 (64.2)		11.1 (1.82)		12.66 (1.34)		13.42 (1.37)		9.25 (1.99)			10.44 (1.49)	11.93 (1.59)	
	Fairly satisfactory	14 (26.4)		11.27 (1.06)		11.67 (1.57)		13.39 (1.29)		8.44 (2.24)			11.59 (1.34)	10.56 (2.2)	
	Rather unsatisfactory	3 (5.7)		10.53 (0.63)		13.01 (0.34)		12.59 (2.48)		7.92 (1.86)			7.35 (0.49)	8.92 (1.09)	
	Very unsatisfactory	2 (3.8)		10.92 (2.71)		11.30 (0.53)		13.34 (0.23)		6.44 (1.18)			9.17 (3.54)	10.8 (0.99)	
	*P* value^a^			.92		.54		.87		.19			*.01*	*.01*	

^a^Italic formatting indicates significant differences among groups.

^b^FM: family medicine.

### Score Evolution

**Figure 4:**
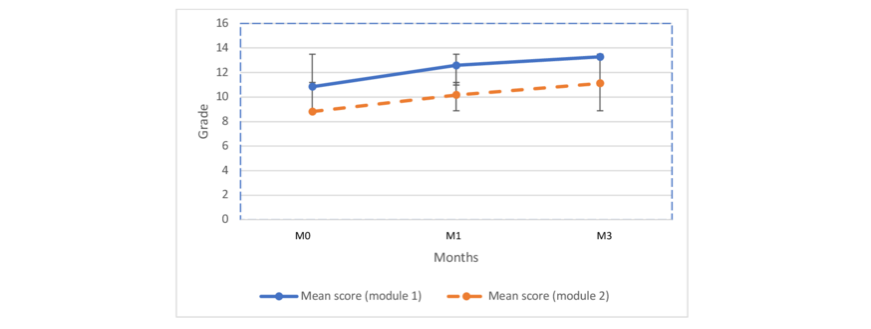
Mean (SD) scores for each e-learning training module over time (assessments at months 0, 1, and 3).

Overall, while we observed some significant differences according to the participants’ profiles at some time points, there was no clear and consistent pattern on the effect of the collected characteristics on their performance. Nevertheless, the residents’ scores improved significantly with increasing assessment time (from 0 to 1 month, 1 month to 3 months, and 0 to 3 months), which is noteworthy (Table 2; Figure 4). In addition, scores increased for up to 92% (n=82) of students for the level 1 module (*P*<.001) and up to 87% (n=55) for the level 2 module (*P*<.001; Table 2; Figure 4).

**Table 2:** Changes in score among residents over time.

Characteristics			Residents with changed scores, n (%)				
			Between months 0 and 1		Between months 1 and 3		Between months 0 and 3
**Module 1**							
	Increased	73 (82)		64 (71.9)		82 (92.1)	
	Decreased	15 (16.9)		22 (25.7)		7 (7.9)	
	*P* value	<.001		<.001		<.001	
**Module 2**							
	Increased	49 (77.8)		43 (68.2)		55 (87.3)	
	Decreased	14 (22.2)		16 (25.4)		8 (12.7)	
	*P* value	<.001		<.001		<.001	

### Satisfaction

Among all participants, 53 (39.6%) trainees filled out the satisfaction questionnaire (Table 1). Using the Likert scale, 90.6% (n=48) found the training very or fairly satisfactory in terms of meeting their expectations. The participants most satisfied with the training had significantly better results at M1 and M3 for module 2.

Twenty-eight (20.9%) trainees answered in the free comments section, including 2 noninformative remarks (“Nothing to report” and “Thank you”). In the end, we analyzed 26 (19.4%) free comments. The top 3 requests made by students in this section were to (1) undertake a face-to-face course to consolidate the e-learning skills (n=14, 26.4%), (2) provide access to ready-made summary sheets (organized by lesion type) and tutorials to learn how to use the equipment (n=6, 11.3%), and (3) provide access to an image bank for training purposes (n=2, 3.8%).

## Discussion

### Principal Findings

The e-learning training course was effective in improving the dermoscopy skills of family medicine residents. Three-month scores greatly improved in 92.1% (n=82) and 87.3% (n=55) of participants for module 1 and module 2, respectively. Dermoscopy training studies often focus on melanoma detection and are expected to improve sensitivity between 13% and 15%, with no improvement in specificity [2]. In the case of multitopic courses such as this study, a published 2-day multitopic dermoscopy training course (melanoma, angioma, seborrheic keratosis, and cutaneous carcinoma) for primary care physicians improved sensitivity by 23.6% and specificity by 21% [20]. Existing short workshops with repeated training sessions (such as this study) showed an improvement in posttraining scores (+21.3%), which correlated with the number of repeated web-based training sessions [21]. They involved a small number of participants (n=27) compared with our 134 participants.

This study also provided some interesting insights about the dermoscopy training of family medicine residents. The ones who reported knowing how to use a dermatoscope before training did not perform any better than others. This is consistent with the fact that providing a dermatoscope without training to all family doctors would not improve skin cancer screening [2,8,10]. Students interested in dermatology and those who had completed a dermatology rotation during their medical school (which probably overlapped) performed better on the pretest of the first module. This difference disappeared after training, suggesting that their dermoscopy input exceeded their previous dermoscopy knowledge. Residents who had completed (or were in the process of completing) a dermatology rotation during their residency also performed better on the assessment at M1 of module 1 and M3 of module 2. In this study, we did not differentiate between residents who were currently completing a dermatology rotation and those who had completed one, nor did we differentiate between the type of rotation (inpatient or outpatient). Subject to further study, the lack of superiority of these students in the pretest may be interpreted as a need for time and practice beyond their rotation experience. As for their superiority at M1, but only for module 1, and at M3 for module 2 (the more complex of the 2 modules), this could show that their rotation potentiates their long-term results and confirms the interest of mixed training. We hypothesized that a replacement activity in family medicine would have a positive effect on their results as consultations related to the cutaneous system are common, estimated at 5.5% of family medicine consultations [22]. Indeed, those who reported seeing patients frequently for dermatologic causes had better results in module 1. This may reflect a higher level of investment and interest of these residents, who may be more aware of local needs.

Most of the participants (n=48, 90.6%) were satisfied with the course and 98.1% (n=52) would recommend it to their peers. Their confidence also increased over time. At the end of the course, 88.7% (n=47) declared that their dermatology skills had improved, which is consistent with the literature [20]. These excellent results are even more important since the level of satisfaction had an impact on our participants’ scores. This process is thus part of a virtuous cycle: the more satisfied students are with a course, the more time and attention they will devote to it, resulting in more effective learning and skill acquisition. One of our most interesting results was that 96.2% (n=51) of participants said they would consider using a dermatoscope in their future practice, closely reflecting the literature [3]. Practicing general practitioners have shared that they are prepared to commit up to 7 days of their personal time to training in dermatoscopy [11].

To our knowledge, this is the first study to focus on dermoscopy training for FM residents. It also benefited from an excellent satisfaction rate, although it had been conducted in the context of the COVID-19 pandemic thanks to the use of e-learning.

### Limitations

We had to make some decisions that introduced potential bias. Recruitment was voluntary, a source of selection bias. Participants were generally the most interested in the subject and the most committed, which led to better results [23]. We also excluded first-year residents, as this training is optional and therefore only accessible to second- and third-year family medicine residents at Montpellier-Nîmes Faculty of Medicine. Finally, the study was monocentric.

Although the e-learning format was well accepted by the students, there was a significant dropout rate of 33.6% (45/134) at 3 months after the start of the level 1 module and 52.9% (71/134) at 3 months after the start of the level 2 module. This phenomenon could be explained by the fact that the 3-month assessment took place during the summer (and therefore the vacations of some) and by the choice to repeat the e-learning 3 times (which could be a source of fatigue, as expressed by some comments to the study and a source of memorization bias). However, it should be noted that loss of focus rates in dermoscopy training is in the order of 40% or more [19,24].

### Perspectives

The decline over time in the diagnostic performance in dermoscopy among family doctors is well known and could be also noted for family medicine residents [11]. Several digital tools could be deployed for our training purposes. The use of digital portfolios in medical education could guide the development of educational content adapted to each student’s initial level of knowledge, needs, and possible preferences, with a view to personalized e-learning. Adaptive e-learning could lead to an increase in student satisfaction, a progression adapted to their level and preferences, and an overall improvement in their skills by encouraging them to work on topics that they find difficult [25]. This should be considered cautiously as the residents’ self-assessed level of competence in dermatology in this study had no effect on their results. It will be necessary to go beyond simple self-assessment in this area to guide their education. Smartphone apps based on training modules, such as Youdermoscopy, could serve as diagnostic aid tools and help increase the user’s knowledge of dermatology [26]. This course could also be a step toward the establishment of a local network of physicians practicing dermoscopy. It is 1 of the 25 practical recommendations in primary care dermoscopy [27]. family doctors need to be supported in their daily practice, especially through training and the establishment of local networks (eg, in the context of coordinated practice based on teledermoscopy) [28-30]. In the short term, this could help our patients and our overburdened health care system in dermatology. In the medium term, digital tools supporting dermatologic diagnosis are expected to play a major role in benefiting patients, once the challenges for identifying dermoscopic features have been overcome [31,32].

## Abbreviations

FD: family doctor
FM: family medicine

## Data Availability

The data sets generated and analyzed during this study are available from the corresponding author on reasonable request.
